# Mechanisms of a *Mycobacterium tuberculosis* Active Peptide

**DOI:** 10.3390/pharmaceutics15020540

**Published:** 2023-02-06

**Authors:** Komal Umashankar Rao, Ping Li, Charlotte Welinder, Erik Tenland, Pontus Gourdon, Erik Sturegård, James C. S. Ho, Gabriela Godaly

**Affiliations:** 1 Department of Microbiology, Immunology and Glycobiology, Institution of Laboratory Medicine, Lund University, SE-22362 Lund, Sweden; 2 Department of Experimental Medical Science, Lund University, SE-22362 Lund, Sweden; 3 Swedish National Infrastructure for Biological Mass Spectrometry, Lund University, SE-22362 Lund, Sweden; 4 Department of Biomedical Sciences, University of Copenhagen, DK-2100 Copenhagen, Denmark; 5 Department of Clinical Microbiology, Institution of Translational Medicine, Lund University, SE-21428 Malmö, Sweden; 6 Singapore Centre on Environmental Life Sciences Engineering (SCELSE), Nanyang Technological University, Singapore 637553, Singapore

**Keywords:** *Mycobacterium tuberculosis*, antimicrobial peptides, treatment, mechanisms of action

## Abstract

Multidrug-resistant tuberculosis (MDR) continues to pose a threat to public health. Previously, we identified a cationic host defense peptide with activity against *Mycobacterium tuberculosis* in vivo and with a bactericidal effect against MDR *M. tuberculosis* at therapeutic concentrations. To understand the mechanisms of this peptide, we investigated its interactions with live *M. tuberculosis* and liposomes as a model. Peptide interactions with *M. tuberculosis* inner membranes induced tube-shaped membranous structures and massive vesicle formation, thus leading to bubbling cell death and ghost cell formation. Liposomal studies revealed that peptide insertion into inner membranes induced changes in the peptides’ secondary structure and that the membranes were pulled such that they aggregated without permeabilization, suggesting that the peptide has a strong inner membrane affinity. Finally, the peptide targeted essential proteins in *M. tuberculosis*, such as 60 kDa chaperonins and elongation factor Tu, that are involved in mycolic acid synthesis and protein folding, which had an impact on bacterial proliferation. The observed multifaceted targeting provides additional support for the therapeutic potential of this peptide.

## 1. Introduction

Drug-resistant pathogens caused 4.95 million deaths in 2019 [1]. Today, the causative agent of tuberculosis (TB), *Mycobacterium tuberculosis*, kills a person every 22 s and belongs to the six pathogens that are responsible for most of the antimicrobial resistance-associated deaths [1]. TB is a severe infection with extremely long treatment time required to eliminate the infection and to counteract the emergence of antibiotic resistance [2]. The global cure rates for MDR and drug-susceptible TB are 54% and 83%, respectively [3]. Additionally, patients infected with extensively drug-resistant (XDR) *M. tuberculosis* strains, which are resistant to all first-line drugs and all second-line TB drugs, can be untreatable [4]. New TB drugs such as bedaquiline, delamanid and pretomanid are effectively used to treat MDR/XDR infections, but clinical resistance to these compounds was reported less than three years after their introduction [5]. Research for new drug targets is thus highly motivated.

Mycobacteria possess a unique hydrophobic membrane comprising several lipid-enriched layers with a low permeability, distinguishing them from other bacteria. However, as this membrane is essential for mycobacterial survival, it is nevertheless a credible target for novel therapeutics. Antimicrobial peptides (AMPs) are a promising alternative to classical antibiotics in the fight against multi-resistant bacteria. In our screening facility, we previously identified a fungi-derived cationic peptide with activity against several clinical isolates of *M. tuberculosis* and MDR *M. tuberculosis*, drug-resistant *Staphylococcus aureus* (MRSA), and nontuberculous mycobacteria (NTM) [6,7]. This peptide, NZX, is non-toxic and resistant to degradation, and its bactericidal capacity has been evaluated in several murine TB infection models [6,7,8]. These experiments revealed that this peptide eliminated *M. tuberculosis* comparably to rifampicin and had an additive effect with ethambutol.

As a group, AMPs are a highly diverse class of molecules, both in terms of their structures and mechanisms of action. The classification of AMPs is, among other things, based on their amino acid composition that determines their source, potential mechanisms, and potential resistance development [9]. The investigated peptide is a derivative of plectasin, originally isolated from *Pseudoplectania nigrella*, that specifically targets cell wall precursors in *S. aureus* by binding to the peptidoglycan lipid II N-terminal amino groups [10,11]. Lipid II is also the main target of glycopeptide antibiotics, such as vancomycin [12], which have an effect on XDR strains together with cerulenin [13]. We previously analyzed murine lung tissue after treating *M. tuberculosis*-infected mice by gold-labelling this peptide [6]. Using electron microscopy, we found the peptide in alveolar macrophage phagosomes associated with *M. tuberculosis* [6]. In addition, we observed a concentration-dependent reduction in intracellular *M. tuberculosis.* In this study, we investigated the peptide’s interactions with live mycobacteria and a model of bacterial inner membranes to gain more understanding of the bactericidal effect of this peptide.

## 2. Materials and Methods

### 2.1. Peptide

The peptide plectasin NZX (GFGCNGPWSEDDIQCHNHCKSIKGYKGGYCARGGFVCKCY) contains the same cysteine residues as plectasin (GFGCNGPWDEDDMQCHNHCKSIKGYKGGYCAKGGFVCKCY), with disulfide bonds at positions C4–C30, C15–C37, and C19–C39 suggesting structural similarity [6,10]. NZX differs from plectasin with three amino acids at positions D9S, M13I, and K32R. NZX was manufactured by solid-phase peptide synthesis, followed by the cyclisation of three natural occurring di-sulfide bonds and purification by sequential chromatography (PolyPeptide Laboratories AB, Limhamn, Sweden). The purity (>97%) of the peptide was confirmed with high- performance liquid chromatography (Thermo Fisher Scientific, Waltham, MA, USA) [6].

### 2.2. Bacteria

In preparation for electron microscopy studies and growth kinetics assays, H37Rv (ATCC 27294) and two clinical strains isolated from pleural effusions (TB1298 (Mtb 1) and TB4001 (Mtb 2)) were cultured in an MGIT960 culture system (BACTEC MGIT 960, Becton Dickinson, Franklin Lakes, NJ, USA) according to the manufacturer’s instructions. TB4001 is an MDR-TB strain, resistant to rifampicin and isoniazid (Supplementary Table S1). The other clinical strain was fully susceptible to first-line antibiotics, and both were verified to be *M. tuberculosis* using standard methods at Clinical Microbiology, Regional Laboratories Skåne, Lund, Sweden.

For mass spectrometry analysis, the *Mycobacterium bovis* bacillus Calmette–Guerin (BCG) Danish strain (supplied by Prof. B. Robertson, Imperial College, London, UK) was prepared as previously described [14]. Briefly, mycobacteria were grown in a Middlebrook 7H9 broth, supplemented with 10% ADC enrichment (Becton Dickinson, Oxford, UK) and hygromycin (50 mg/L; Roche, Lewes, UK). The culture was washed twice with sterile phosphate-buffered saline (PBS), re-suspended in the broth, and dispensed into vials. Glycerol was added to a final concentration of 25%, and the vials were frozen at −80 °C. Prior to each experiment, a vial was defrosted, added to 9 mL of a 7H9/ADC/hygromycin medium, and incubated with shaking for 72 h at 37 °C. Mycobacteria were then centrifuged for 10 min at 3000× *g*, washed twice with PBS, and re-suspended in 10 mL of PBS.

### 2.3. Growth Kinetics

To assess the effect of the peptide on bacterial growth kinetics against two clinical strains of *M. tuberculosis* (Mtb 1 and 2), we used the MGIT960 culture system (BACTEC MGIT 960, Becton Dickinson, Franklin Lakes, NJ, USA) following previously validated methods [15]. Briefly, the peptide was diluted to 3.2, 6.3 or 12.5 μM in PBS and added to MGIT960 culture tubes. *M. tuberculosis* in the log phase (0.5 McFarland, ~1.5 × 10^8^ CFU/mL) was added to the MGIT960 tubes. Using this culture system, we performed kinetic studies comparing the control strains (without the peptide, 1:10 or 1:100 diluted controls of the corresponding strain) to the peptide-threatened strains. The kinetic studies were determined as the peptide concentration where there was less growth compared with the 1:10 or 1:100 diluted controls (without the peptide) of the corresponding strain, i.e., the lowest concentration that inhibited >99% or >90% of the bacterial population, respectively.

### 2.4. Scanning Electron Microscopy

The effect of the peptide on *M. tuberculosis* H37Rv was determined with scanning electron microscopy (SEM). Bacteria were grown to 1 × 10^8^ CFU and treated with 6.3 μM of the peptide for 0, 0.5, 2, 4 or 24 h. The bacteria were then pelleted at 3000× *g* for 7 min, suspended in a fixation solution (4% formaldehyde and 2.5% glutaraldehyde in sodium cacodylate), and absorbed onto poly-L-lysine-coated glass coverslips for 1 h. Samples were processed as previously described [16] and examined with a Philips/FEI XL30 FEG scanning electron microscope (Philips, Lund, Sweden) at an acceleration voltage of 5 kV and a working distance of 10 mm.

### 2.5. Transmission Electron Microscopy

For transmission electron microscopy (TEM) (Philips, Lund, Sweden) and the visualization of the peptide’s effects on bacteria, the *M. tuberculosis* strain H37Rv (1–2 × 10^6^ CFU/sample) was incubated for 0, 0.5, 1, 4 or 24 h at 37 °C with the peptide (6.3 μM), which was pre-labeled with 5 nm of colloidal gold. Gold-labeled untreated *M. tuberculosis* H37Rv was used as the respective control.

Bacterial samples were adsorbed onto carbon-coated copper grids for 2 min, briefly washed with two drops of water, and negatively stained with two drops of 0.75% uranyl formate. The grids were rendered hydrophilic by glow discharge at a low pressure in air. All samples were examined with a Jeol JEM 1230 electron microscope operated at an 80 kV accelerating voltage. Images were recorded with a Gatan Multiscan 791 charge-coupled device camera (Gatan, Pleasanton, CA, USA).

### 2.6. Preparation of Large Unilamellar Vesicles

To mimic complex bacterial inner membranes, we produced large unilamellar vesicles consisting of binary 1-palmitoyl-2-oleoyl-*sn*-glycero-3-phosphoethanolamine (POPE) and 1-palmitoyl-2-oleoyl-*sn*-glycero-3[phospho-rac-(1-glycerol) (POPG). The required amounts of POPE and POPG in chloroform were mixed at molar ratios of 30:70 or 85:15 in round-bottom glass tubes to mimic an *Escherichia coli* inner membrane and, as a comparison, a Gram-positive bacterial-mimicking lipid membrane, respectively [17]. The mixture was dried using a stable stream of nitrogen gas to form thin lipid films, followed by extended drying in a vacuum desiccator for up to 3 h. Unless otherwise stated, each dry lipid film was rehydrated in phosphate-buffered saline (PBS) at 37 °C to first form a 2 mg/mL multilamellar vesicle suspension, which was then resized by extrusion (21 times) on an Avanti Mini Extruder assembled with a 100 nm polycarbonate membrane. For the vesicle leakage assay, the dry lipid films were rehydrated in 50 mM of calcein (in PBS; Sigma, Lund, Sweden), extruded, and dialyzed overnight to obtain vesicles encapsulating calcein at a self-quenching concentration. For the vesicles used for the fluorescence microscopy experiments, 1,2-dipalmitoyl-*sn*-glycero-3-phosphoethanolamine-*N*-(lissamine rhodamine B sulfonyl) (Rhod-PE) or 1,2-dipalmitoyl-sn-glycero-3-phosphoethanolamine-N-(7-nitro-2-1,3-benzoxadiazol-4-yl) (NBD-PE) was added to the initial chloroform mixture at 0.1 mol%. The vesicles were stored at 4 °C until usage. All lipids were purchased from Avanti Polar Lipids (Ascent Building, Singapore).

### 2.7. Circular Dichroism Spectroscopy

Circular dichroism (CD) spectra were used to identify changes in the peptide’s secondary structure upon vesicle interaction. CD spectra were measured on an AVIV 420 Circular Dichroism (Aviv Biomedical Inc., New York, NJ, USA) spectrometer. Measurements were performed in duplicate at 37 °C in a quartz sandwich cuvette with an optical path length of 0.5 mm at a constant peptide concentration of 33 µM. Data were acquired over a wavelength range of 190–260 nm with 1.0 nm wavelength steps, an averaging time of 0.1 s, and a 1.00 nm bandwidth, and readings were averaged over three scans. Background correction was performed by measuring buffer spectra containing the peptide. The secondary structure content was estimated using the K2D3 algorithm [18]. Data points from 195t o 240 nm were used for the analysis. The spectra were smoothed at 12 pts via the adjacent-averaging method and plotted via OriginPro 9.1 (Origin Lab, Northampton, MA, USA).

### 2.8. Calcein Experiment

Calcein-dye-encapsulated vesicles (PMV and NMV) were transferred to a 96-well plate and incubated with the peptide at concentrations ranging from 625 nM to 22 mM, with two technical replicates per concentration. The experiments were repeated four times. The samples were excited at 480 nm, and fluorescence emissions at 520 nm were monitored over 1 h prior to the lysis of vesicles with Triton X-100 (0.05%) using TECAN infinite^®^ M200 PRO (Tecan, Männedorf, Switzerland).

### 2.9. Dynamic Light Scattering

Two concentrations of the peptide, 3 mM and 33 mM, were mixed with the extruded vesicles (PMV and NMV). All samples were aliquoted into disposable cuvettes. Dynamic light scattering (DLS) was performed with a Malvern Zetasizer Nano ZS. An average of 33 runs (10 s per run) was collected using a 173° detector. Time-dependent aggregation kinetics were observed for about 30 min when vesicles were mixed with the peptide, measured at room temperature. Data were plotted in Microsoft Excel (16.69.1).

### 2.10. Intrinsic Tyrosine and Tryptophan Fluorescence Measurement

Peptide interactions with PMV and NMV were analyzed by collecting the intrinsic fluorescence emissions of the tryptophan (Trp) and tyrosine (Tyr) residues. Tyr and Tyr/Trp fluorescence intensities were read at 295 nm and 280 nm, respectively, with a microplate reader (TECAN infinite^®^ M200 PRO) from a 96-well plate containing the peptide and vesicles up to a total volume of 200 μL at room temperature. Auto-fluorescence from the peptide and vesicles was corrected prior to analysis.

### 2.11. Labelling and Co-Immunoprecipitation of the Peptide

To label the peptide in preparation for the LC–MS/MS studies, the NZX gene was synthesized from Genescript (Rijswijk, Netherlands). A 6×His tag with a TEV cleavage site was fused to the N-terminus of the peptide and cloned into a pET-22b vector. The pET-22b-6×His-TEV-NZX expression plasmid was transformed into the *E. coli* strain BL21(DE3) cells (Thermofisher). A single colony was incubated in 10 mL of a Luria broth (LB) medium supplied with 100 mg/L of ampicillin and cultured at 37 °C overnight. The pre-culture was transferred into a LB medium with 100 mg/L of ampicillin and incubated for 3 h at 37 °C with shaking at 180 rpm. Protein production was induced at final concentration of 1 mM of IPTG when the OD reached approximately 0.6, and then we continued culturing at 37 °C for 4 h. The cells were harvested by centrifugation at 5000× *g* for 30 min. The cell pellets were washed with 30 mL of a 1×PBS buffer and then re-suspended the 1×PBS buffer supplied with 5% glycerol, 2 mM of MgCl_2_, and 0.01 mg/mL of DNase I (100 mg/mL). The cells were disrupted by sonication for 20 min. Unbroken cells and cell debris were removed by centrifugation at 38,000× *g* for 30 min. The supernatant was filtered (0.2 μm) and applied to an Ni-NTA resin for affinity purification. The column was washed with 50 mL of the 1×PBS buffer supplied with 5% glycerol and 20 mM of imidazole, and then the column was washed again with 30 mL of the 1×PBS buffer supplied with 5% glycerol and 40 mM of imidazole. The protein was eluted with 10 mL of an elution buffer (1×PBS, 5% glycerol, and 300 mM of imidazole). The eluted protein sample was checked with SDS-PAGE and identified with mass spectrometry.

For co-immunoprecipitation (Co-IP), two bed volumes of His-Pur Ni-NTA resin (Thermo Scientific, Lund, Sweden) were equilibrated using an equilibration buffer, 20 mM of Na_3_PO_4_, and 300 mM of NaCl. We then mixed 10 μg of the his-tagged peptide with resins for 1 h on an end-over-end rotator. Resins were washed with two bed volumes of 1×PBS (washing buffer) and re-incubated with 100 μg of whole-cell lysate of the BCG grown to an O.D_600_ of 0.5 for 1 h. The resins were washed to remove non-specific proteins, and bound protein complexes were eluted by adding 250 mM of imidazole (Sigma-Aldrich, Lund, Sweden).

As a control, NZX was also labeled with biotin at the C-terminal through synthesis using a mild Fmoc chemistry method (Mimitopes, Victoria, Australia). Co-precipitation experiments were performed using a streptavidin pierce MS-compatible magnetic IP kit (Thermo Scientific). Premixed biotinylated antibodies and cell lysates in an IP-MS cell lysis buffer was gently vortexed with 25 μL of streptavidin magnetic beads. The beads were washed with a 175 μL cell lysis buffer (×2) and incubated with biotin-labeled NZX at room temperature for an hour in an end-over-end rotator. The unbound sample was removed, and the beads were further washed with IP-MS wash buffers A and B from the kit. Protein complexes bound to the beads were eluted with 100 μL of the IP-MS elution buffer.

### 2.12. Sample Preparation for LC–MS/MS

Samples (50 µL) were added to Mini Bio-Spin Chromatography Columns (#7326207, BioRad, Solna, Sweden) for the removal of the remaining resins from the Co-IP and spun 250× *g* for 2 min. The resin was washed with 200 µL of 100 mM ammonium bicarbonate. The samples were reduced with dithiothreitol to a final concentration of 10 mM and heated at 56 °C for 30 min, followed by alkylation with iodoacetamide to a final concentration of 20 mM for 30 min at room temperature in the dark. Digestion was performed by adding trypsin in a ratio of 1:50 (Sequencing Grade Modified Trypsin, Part No. V511A, Promega, Nacka, Sweden) to the samples, which were incubated overnight at 37 °C. The digestion was stopped with 20 µL of 10% trifluoroacetic acid. The peptides were placed in a Speed Vac to dry them, and they were resolved in 2% ACN/0.1% TFA.

### 2.13. Mass Spectrometry Acquisition

The LC–MS/MS analysis was performed using a Tribrid mass spectrometer fusion equipped with a Nanospray Flex ion source and coupled with an EASY-nLC 1000 ultrahigh pressure liquid chromatography (UHPLC) pump (Thermo Fischer Scientific, Waltham, MA, USA) [19]. The proteins, 1 µg, were injected into the LC–MS/MS system. Proteins were concentrated on an Acclaim PepMap 100 C18 precolumn (75 μm × 2 cm, Thermo Scientific, Waltham, MA, USA) and then separated on an Acclaim PepMap RSLC column (75 μm × 25 cm, nanoViper, C18, 2 μm, 100 Å) at a temperature of 40 °C and a flow rate of 300 nL/min. Solvent A (0.1% formic acid in water) and solvent B (0.1% formic acid in acetonitrile) were used to create a nonlinear gradient to elute the peptides. For the gradient, the percentage of solvent B was maintained at 3% for 3 min, increased from 3% to 25% for 60 min, increased to 60% for 10 min, increased to 90% for 2 min, and then kept at 90% for another 8 min to wash the column.

The Orbitrap Fusion (ThermoFisher, Waltham, MA, USA) was operated in the positive data-dependent acquisition (DDA) mode. The peptides were introduced into the HPLC–MS via a stainless-steel nano-bore emitter (OD 150 µm, ID 30 µm) with a spray voltage of 2 kV and a capillary temperature of 275 °C. Full MS survey scans from *m*/*z* 350 to 1350 with a resolution of 120,000 were performed in the Orbitrap detector. The automatic gain control (AGC) target was set to 4 × 10^5^ with an injection time of 50 ms. The most intense ions (up to 20) with charge states of 2–5 from the full scan MS were selected for fragmentation in the Orbitrap. The MS2 precursors were isolated with a quadrupole mass filter set to a width of 1.2 *m*/*z*. Precursors were fragmented with high-energy collision dissociation (HCD) at a normalized collision energy (NCE) of 30%. The resolution was fixed at 30,000, and for the MS/MS scans, the values for the AGC target and injection time were 5 × 10^4^ and 54 ms, respectively. The duration of dynamic exclusion was set to 45 s, and the mass tolerance window was 10 ppm.

The raw DDA data were analyzed with Proteome Discoverer™ 2.5 Software (Thermo Fisher Scientific, Waltham, MA, USA). The peptides were identified using both SEQUEST HT and Mascot against the UniProtKB Mycobacterium strain with the BCG/Pasteur 1173P2 database (UP000001472). Due to the low number of PSMs in the data search, the software could not calculate any mass corrections. Therefore, a contamination database was downloaded from www.matrixscience.com (http://www.matrixscience.com/help/seq_db_setup_contaminants.htm, accessed on 13 August 2019) and added in the search. The search was performed with the following parameters: N-terminal acetylation as dynamic modifications and cysteine carbamidomethylation as static modification. Precursor tolerance was set to 10 ppm, and fragment tolerance was set to 0.05 ppm. Up to 2 missed cleavages were allowed, and Percolator was used for peptide validation at a maximum q-value of 0.05. The extracted peptides were identified and quantified via label-free relative quantification. The extracted chromatographic intensities were used to compare protein abundance across samples.

## 3. Results

### 3.1. The Peptide Interacts with M. tuberculosis Membrane

**Figure 1:**
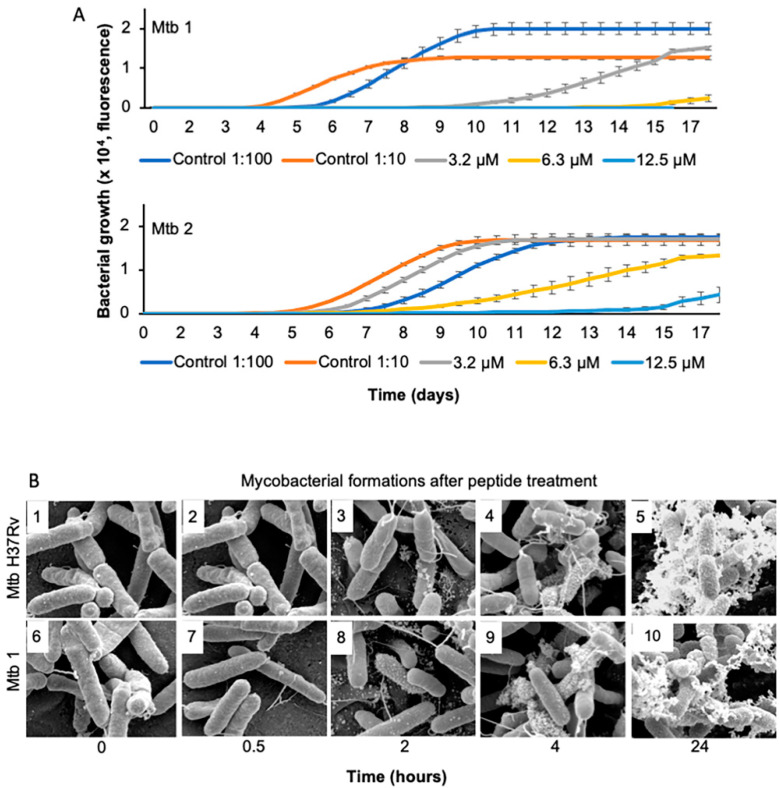
Kinetics of cell proliferation and death in clinical *M. tuberculosis* strains upon exposure to NZX. (**A**) Graphs depicting the growth kinetics of *M. tuberculosis* using the MGIT960 culture system for a period of 18 days. The *M. tuberculosis* isolates Mtb 1 and Mtb 2 were treated once with 3.2, 6.3 or 12.5 μM NZX at day 0, and their growth was measured for 18 days. Data depict an average of three replicates. (**B**) *M. tuberculosis* H37Rv and the clinical *M. tuberculosis* isolate Mtb 1 were treated with 6.3 μM of NZX for up to 24 h and visualized with scanning electron microscopy (SEM). Different kinds of destabilization of the membrane were observed, from intact forms to bubbling formations. Scale bar: 2 μm. Experiments were repeated three times for each strain.

The influence of the peptide on mycobacterial growth was studied by analyzing the growth kinetics of two clinical *M. tuberculosis* isolates treated with a single dose of the peptide (Figure 1A). The treatment induced a concentration-dependent reduction in initial bacterial growth and a long-term effect on both bacterial populations. A peptide concentration of 12.5 μM eliminated Mtb 1, while the MDR-resistant isolate Mtb 2 required higher concentrations to show the same pattern. Peptide interactions with the mycobacterial surface was further studied using scanning electron microscopy (Figure 1B). *M. tuberculosis* H37Rv and the clinical isolate Mtb 1 were treated with 6.3 µM of the peptide. Untreated bacteria appeared characteristically rod-shaped and smooth (Figure 1B(1 and 6)). The addition of the peptide triggered membranous protrusions and tube-shaped structures on both bacterial surfaces (Figure 1B). Increased bubble-like structures were observed from 30 min of treatment with the peptide (Figure 1B(2 and 7)). These structures progressively appeared in all bacteria (Figure 1B(3–4 and 8–9)). These features progressed to a massive bubbling morphology, and the time scale of its emergence was correlated with death, as shown by the samples 24 h after peptide treatment (Figure 1B(5 and 10)). After further analyzing the structural morphology (below), we found a high resemblance to membrane vesicles [20,21,22,23].

### 3.2. The Peptide Affects Mycobacterial Inner Membrane

**Figure 2:**
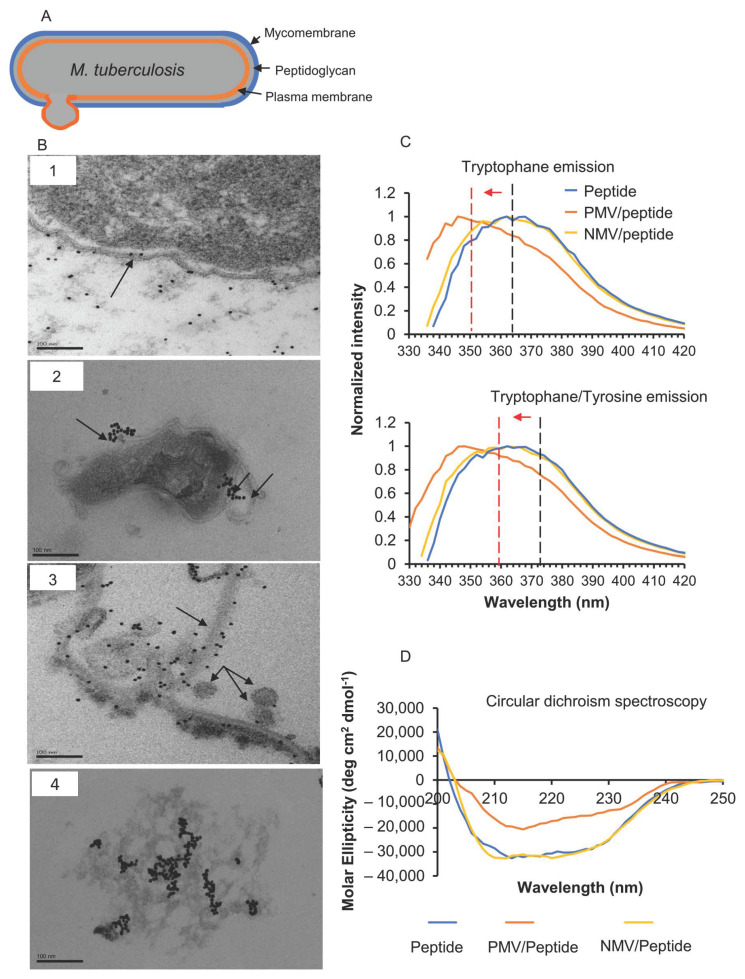
NZX characteristics upon binding. (**A**) Simplified model of the organization of the mycobacterial envelope. (**B**) Transmission electron microscopy (TEM) showing the gold-labeled NZX that caused the membrane destabilization of *M. tuberculosis* (1–4). Scale bar: 100 nm. (**C**) Representative graph of intrinsic Trp + Tyr (upper panel) and Trp (lower panel) emission spectra of the peptide when interacting with the LUV at pH 7.4. In the presence and absence of vesicles, shifts in wavelength (arrow) and increased fluorescence intensity were recorded. (**D**) CD structure of NZX in the presence of vesicles. Peptide in PBS was used as the control. Secondary structure content was analyzed with the K2D3 server.

Transmission electron microscopy (TEM) and a gold-labeled peptide were used to elucidate the structural changes in *M. tuberculosis* caused by the peptide treatment. Within one hour of exposure, the peptide was found to be associated with the *M. tuberculosis* H37Rv outer mycomembrane (Figure 2B(1)) and a disruption of the plasma membrane was observed (Figure 2B(2, arrow)). Initially, the peptide appeared to be traversing through the bacterial mycomembrane without causing any visible damage. Next, the peptide was found to be in the plasma membrane through what appeared to be an invagination process (Figure 2B(2, two arrows)) [24]. In the plasma membrane, peptide aggregates were found to be associated with membrane disruptions (Figure 2B(3)). Alongside the pulled, disassociated membranes, small and detached circular vesicle-like formations (Figure 2B(3, arrows)), as well as less electrodense cells resembling ghost cell (Figure 2B(4)), were observed.

### 3.3. Interfacial Binding of the Peptide to Lipid Membranes

The striking membrane remodeling effect of the peptide on the mycobacterial plasma membrane suggested a mechanism targeting the lipid membrane. Phosphatidylethanolamine (PE) and negatively charged lipids, such as phosphatidylinositol, diacylglycerol, phosphatidylserine, and phosphatidylglycerol, are joint plasma membrane structures of mycobacteria and various Gram-positive bacteria [17,25,26,27,28]. As it is commonly mediated by electrostatic attractive forces and insertion into the hydrophobic moiety of the lipid bilayer, we examined the interactions of the peptide with model membranes comprising 1-palmitoyl-2-oleoyl-*sn*-glycero-3-phosphoethanolamine (POPE) and 1-palmitoyl-2-oleoyl-*sn*-glycero-3[phospho-rac-(1-glycerol) (POPG) as a mimic of a negatively charged bacterial membrane. Two types of 100 nm lipid vesicles—POPE/POPG (30/70, mol/mol, termed PMV) and POPE/POPG (85/15, mol/mol, termed NMV)—were prepared, and the intrinsic fluorescence of the tryptophan and tyrosine residues present in the peptide was measured with or without the vesicles (Figure 2C). The peptide was found to have one tryptophan residue (W8) and three tyrosine residues (Y25, Y29, and Y40) in its sequence. Reductions in the polarity of the environment around tryptophan (λ_ex_ = 295 nm) or the tryptophan/tyrosine residues (λ_ex_ = 280 nm) caused blue shifts in the emission maxima, accompanied by an increase in the quantum yield. Tryptophan and tryptophan/tyrosine emission spectra in the presence of PMV showed a strong blue shift of >10 nm with an increase in fluorescence intensity (Figure 2C). The emission peak of the tryptophan/tyrosine spectrum for the peptide was 360–364 nm and shifted to 346–350 nm in the presence of PMVs. Similar shifts in the emission maximum were observed in the tryptophan spectrum from 360–364 nm to 344–348 nm following PMV addition. These results indicate that these two aromatic residues, predominantly W8, were exposed to a more hydrophobic environment, likely anchoring at the headgroup-acyl tail interfacial region. The addition of NMV, with a lower ratio of negatively charged PG headgroups, to the peptide did not induce a shift or intensity increase in the two emission spectra, suggesting that charge–charge interactions are important for membrane insertion. The non-normalized spectra can be found in Supplementary Figure S2.

### 3.4. Changes in Peptide Secondary Structure upon Interaction with Gram-Positive Membranes

The study of the peptide secondary structure upon its interaction with lipid vesicles was performed with far-UV circular dichroism (CD) spectroscopy (Figure 2D). The peptide alone showed prominent minima around 208 and 222 nm, which suggest a largely alpha helical fold. When mixed with PMVs, the magnitudes of the minima were reduced. Secondary structure content analysis with the K2D3 algorithm [18] estimated a reduction of about 30% in alpha helical content (from 72% to 40%), along with the formation of a beta strand (16%). The CD spectrum of the NMV–peptide mixture was virtually identical to that of the peptide alone.

The results suggest that the peptide anchored to the PMV membrane, which was highly positively charged, and the membrane insertion led to a partial transition of alpha helical fold to beta strand conformation.

### 3.5. Peptide Interactions Do Not Rupture the Membrane

**Figure 3:**
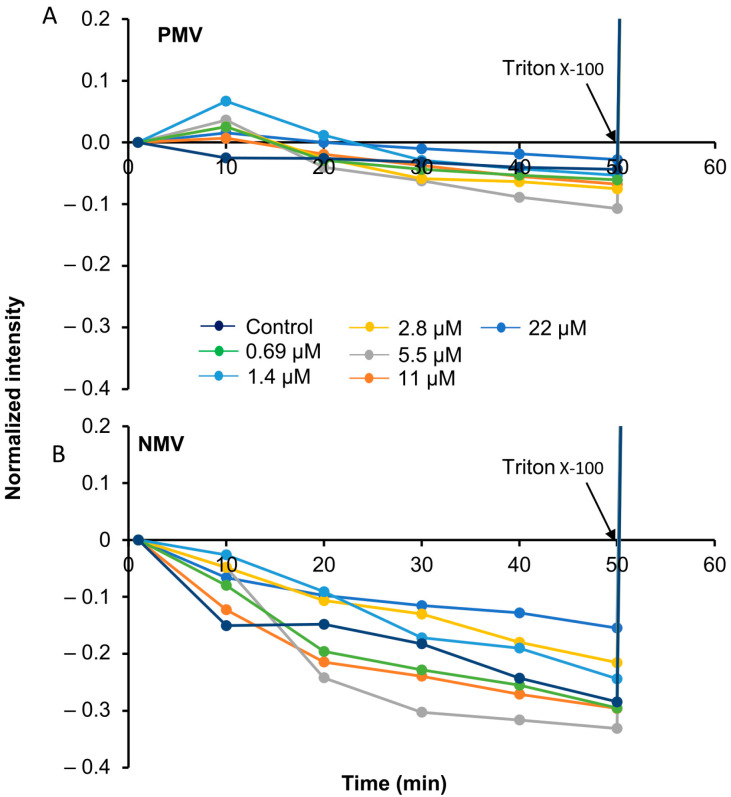
Interactive studies of the peptide with lipid membrane and the permeabilization effect of the peptide through carboxyfluorescein release in the LUV. (**A**) showing PMV and (**B**) showing NMV. The fluorescent intensity from vesicles was recorded for different concentrations of the peptide, incubated over an hour. A representative graph is depicted; data were acquired from a minimum of 3 experiments.

Next, we sought to examine the effect of the peptide on lipid bilayer permeability. PMVs and NMVs loaded with a self-quenching concentration of calcein were incubated with the peptide, and fluorescence intensity was recorded (Figure 3). Membrane permeabilization leading to release of the encapsulated content relieved calcein from the self-quenched environment, leading to an increase in fluorescence intensity. The addition of the peptide (0.69 to 22 μM) to the vesicles did not result in increases in the fluorescence signal. Instead, we noticed a concentration-dependent drop in fluorescence. A gradual decrease in fluorescence intensity was also observed in the control, where this phenomenon is known to be due to a photobleaching effect [29]. The addition of Triton at the end of the experiment resulted in total calcein release, confirming that no membrane rupture was caused by the peptide.

### 3.6. The Peptide Causes Membrane Aggregations

**Figure 4:**
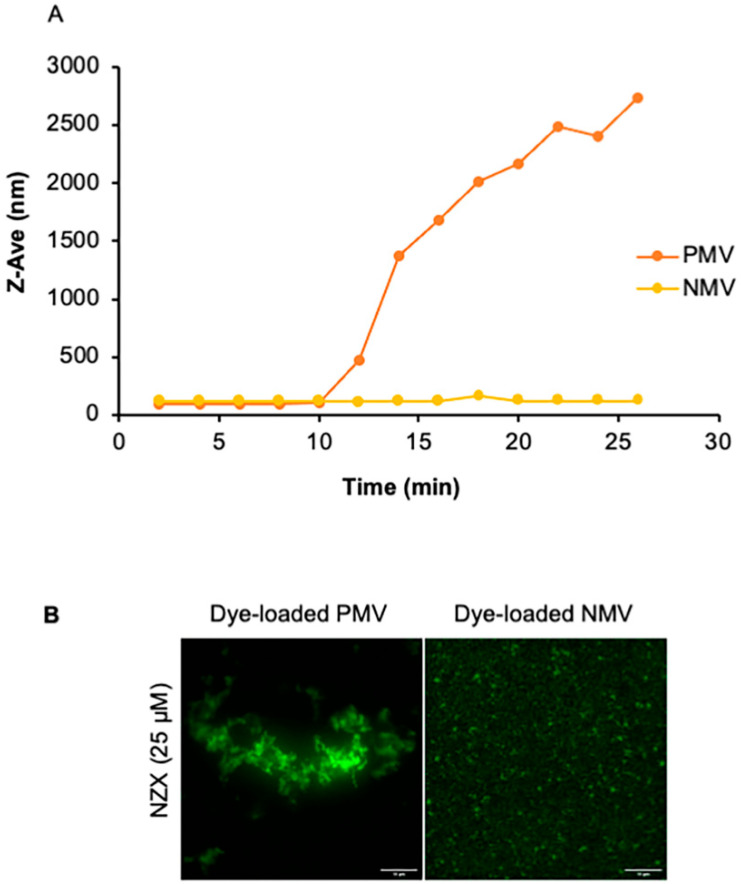
Aggregative properties of the peptide. (**A**) DLS size profiles of the LUV when incubated with NZX. Data show kinetic measurement over thirty minutes in the presence and absence of NZX. (**B**) Representative image of fluorescence microscopy experiments showing the effect of NZX on the LUV at 25 °C. Following the addition of NZX, a rapid vesicle aggregation was observed for the G+ and G- vesicles along with a reduced background signal. After thirty minutes, no further aggregation occurred and the structures remained stable. PMV aggregation was seen to be more rapid and larger in size compared with NMV. Scale bar: 10 μm. Data were acquired from three experiments.

To examine if the decrease in fluorescence was correlated to changes in vesicle size, dynamic light scattering (DLS) was performed. Kinetic measurements were performed on both PMV and NMV over a time course of 30 min (Figure 4A). The addition of 3 μM of the peptide to PMVs gradually increased the Z-average hydrodynamic diameter (Z-ave), along with the polydispersity index (PdI). Increasing the peptide concentration to 25–33 μM resulted in the stochastic fluctuation of D_h_ and PdI of the PMVs (not shown). In comparison, D_h_ and PdI were largely unchanged for the low (3 μM) peptide concentration in NMVs. However, increasing the peptide concentration to 25–33 μM induced stochastic fluctuations in D_h_ and PdI, albeit with much lower magnitudes than those of the PMVs (not shown). These results suggest that additional factors mediate peptide–membrane interactions, which promote vesicle aggregation, in addition to the electrostatic interactions between positively charged residues of the peptide and the negatively charged lipid headgroup.

To further investigate peptide interactions, we conducted the time-lapse fluorescence microscopy of the peptide-vesicle mixtures using PMVs and NMVs with calcein encapsulated at 0.2–1 mM. The background fluorescence appeared to be largely homogeneous, with randomly moving brighter speckles that were consistent with the presence of vesicles that were below the diffraction limit of light and whose movements were governed by random Brownian motion (Figure 4B and Supplementary Figure S3). The addition of the peptide induced the formation of micrometer-sized aggregates with bright fluorescence, exhibiting a bead-on-a-string morphology, along with a decrease in the background fluorescence (Figure 4B). The rate and degree of aggregate formation were higher for PMVs than for NMVs, consistent with the timescale in the DLS measurements (Figure 4A). After 30 min, independent of the vesicle charge, the clumps were stable, with no further size growth. These results confirmed that the peptide caused vesicles to aggregate, supporting the strong membrane affinity of the peptide and consequently promoting close membrane–membrane juxtaposition without permeabilizing the membrane.

### 3.7. The Peptide Targets Essential Proteins in Mycobacteria

We used co-immunoprecipitation and LC–MS/MS to identify possible surface or intracellular proteins targeted by the peptide upon mycobacterial interaction (Table 1). Briefly, a His- or Strep-tagged peptide was incubated with the whole-cell lysate of BCG for 1 h, and then the peptide-bound fraction was eluted with imidazole and subjected to LC–MS analysis. Both the His- and Strep-tag labeling methods gave the same protein profiles, identifying proteins involved in maturation and mycolic acid synthesis, i.e., Cpn1, Cpn2, EF-Tu and acyl carrier protein (AcpM). A hitherto unidentified conserved protein (Rv3269) was also targeted by the peptide (Table 1). These results revealed that the peptide possibly interferes with essential proteins affecting cell survival, including a protein involved in cell envelope formation in *M. tuberculosis*. However, as these results were obtained by incubating the peptide with mycobacterial cell lysate, we do not know if these interactions occur in vivo.

## 4. Discussion

Previously, we demonstrated that this serum-stable peptide eliminated *M. tuberculosis* comparably to rifampicin and isoniazid in vivo [6,7,8]. In this study, we discovered that one dose of NZX to *M. tuberculosis* induced an early concentration-dependent reduction in bacterial growth, followed by a long-term effect on mycobacterial survival. To analyze the peptide’s mechanisms, we used live mycobacteria and biophysical methods to reveal the possible ways that the peptide can kill bacteria. Early interactions on *M. tuberculosis* caused the remodeling of the outer mycomembrane and association with the plasma membrane, which ultimately led to less electron-dense formations of the ghost cell type [30]. Liposome inner membrane studies revealed that peptide interactions caused membrane aggregation and induced confirmational changes in the peptide’s secondary structures. Finally, by binding to essential proteins in the cytoplasm, such as 60 kDa chaperonin 1, elongation factor Tu, 60 kDa chaperonin 2, and acyl carrier protein (AcpM), the peptide also interfered with bacterial mycolic acid and protein synthesis, which may explain the previously observed long-term effect on the mycobacterial population.

As an amphipathic small cationic host defense peptide with a net positive charge at physiological pH, the initial effects of the peptide on mycobacteria were probably driven by electrostatic interactions and hydrophobic effects [10,31]. Electron microscopy revealed membrane remodeling as the peptide crossed the outer membrane, possibly by invagination, i.e., separating and clustering the lipid domains, as described previously [26]. To study the membrane interactions, we used unilamellar liposomes and analyzed the peptide tryptophane (Trp) content due to its effect on bacterial anchoring ability [32]. This hydrophobic moiety was at position 8 to the N-terminus on the investigated peptide. Peptide binding to the liposomal membranes induced a shift, indicating that Trp is important for peptide insertion into bacterial membranes. Inside the cytoplasm, the peptide was found in the vicinity of the condensed chromosome, but whether the effect on nucleoid was stress-related or caused by the direct binding of the peptide to the chromosome is not known [33,34].

Under normal conditions, mycobacteria produce inner membrane vesicles as membrane blebbing, but stress impacting the cell wall, such as antibiotic treatment, increases the expression and may lead to membrane damage and so-called bubbling cell death by forming inner membrane vesicles (IMVs) [20,21,22]. Shortly after peptide treatment, we observed bubbling on the mycobacterial surface that covered the entire bacteria over time, possibly of IMV origin. Studies on charged inner membrane liposomes revealed that the peptide caused concentration-dependent aggregation, showing that the peptide possesses a strong membrane affinity. Interestingly, the affinity was found to be significantly stronger towards Gram-positive membranes than Gram-negative membranes. In a recent study investigating the interactions of an amphiphilic cationic peptide with liposomes in different phases, liposome aggregation was also observed [35]. After analyzing the structure with time-lapse fluorescence microscopy, we found that the formed aggregates presented a bead-on-a-string morphology [23]. This morphology is well-known in apoptosis studies, where the generated extracellular vesicles mediate intracellular communication [36]. However, to our knowledge, this phenomenon has not been reported for AMPs.

AMP amino acid composition determines their potential mechanisms of antibacterial action. The NZX peptide is rich in non-polar glycine amino acids that could activate phagocyte-mediated microbicidal mechanisms, as previously shown with a glycine-rich salmonid peptide [37]. NZX also contains proline residues that could interfere with the initial phase of protein synthesis, as shown for other bacteria [38,39]. Supporting these observations, we could see that the peptide was targeting enzymes involved in mycobacterial protein machinery. Mycobacteria were the first bacteria shown to have multiple chaperonin genes, namely cpn60.1 and cpn60.2 [40,41]. The deletion of cpn60.2 (groEL) in mycobacteria had a direct impact on mycobacterial cell survival due to its essential ability to correctly fold critical housekeeping genes [42,43,44]. Cpn60.2 is further known to be a potent immunogen and has been used as a part of a DNA vaccine against *M. tuberculosis* [45]. Cpn60.1 was recently found to fold key enzymes involved in the synthesis of mycolic acid, and it is physically associated with KasA, a key component of the type II fatty acid synthase involved in mycolic acid synthesis [42,44]. The meromycolate extension acyl carrier protein (AcpM) of *M. tuberculosis* is also known to be involved in mycolic acid biosynthesis, while HCP (Rv3269), a probable heat shock protein in mycobacteria, is involved in oxidative stress [46]. Furthermore, while Cpn60.1 binds with TLR4 and possesses a domain for binding with the CD14 receptor, Cpn60.2 can bind to both TLR2 and TLR4 and has an affinity for the CD43 receptor on macrophages [47,48,49]. Mycobacterial elongation factor Tu (EF-Tu) acts as a GTPase by delivering aminoacyl-tRNA to ribosomal A site [50], while NZX is bound to the rest. Thus, by targeting essential proteins, the peptide could affect a mycobacterial population over a long period, as supported by our previous observations [6].

In summary, our studied peptide possesses multiple ways to target mycobacteria, partly through bactericidal interactions and partly by affecting a colony for long period. Peptide interactions with bacterial membranes induced massive vesicle formation, leading to bacterial cell collapse. In investigating this phenomenon in liposomes, we found further support for the occurrence of strong lipid membrane interactions because the peptide that was inserted into the membrane changed its secondary structure and pulled the liposomes such that they aggregated into a distinct pattern, suggesting that the peptide has a strong inner membrane affinity. This step is probably driven by electrostatic interactions and hydrophobic effects [10,31]. Regarding long-term effects, the peptide seems to target multiple essential enzymes involved in mycolic acid synthesis that could impact bacterial proliferation. The observed possible multi-targeting could be beneficial in terms of resistance development [51,52], further supporting the therapeutic potential of this peptide.

## Figures and Tables

**Table 1:** Identified interacting proteins from Co-IP and LC–MS/MS analysis.

Protein Name	Coverage %	# Proteins	# PSM	# Unique Peptides
Cpn 60.1	44	16	31	15
EF-Tu	48	12	28	11
Cpn 60.2	31	11	18	9
AcpM	24	2	3	2
HCP	29	2	8	2

# = number.

## Data Availability

The raw data are available from the lead contact upon request.

## Supplementary Materials

The following supporting information can be downloaded at: https://www.mdpi.com/article/10.3390/pharmaceutics15020540/s1, Table S1: Antibiotic resistance profile of the clinical MDR isolate; Figure S1: Confirmation of recombinant NZX; Figure S2: The non-normalized spectra; Figure S3: A control image with liposomes without the peptide.
