# Supplementary material for: Mechanisms of a Mycobacterium tuberculosis Active Peptide
Source: Pharmaceutics. 2023 Feb 6;15(2):540. doi: 10.3390/pharmaceutics15020540 (PMC9958537; doi:10.3390/pharmaceutics15020540)
Supplement: Supplementary file 1 [file pharmaceutics-15-00540-s001.zip › pharmaceutics-2182065-supplementary.pdf]

pharmaceutics

Supplementary Materials: Mechanisms of a *Mycobacterium tuberculosis* Active Peptide

Komal Umashankar Rao, Ping Li, Charlotte Welinder, Erik Tenland, Pontus Gourdon, Erik Sturegård, James C. S. Ho and Gabriela Godaly

Table S1. Antibiotic resistance profile of the clinical MDR isolate.

Antibiotic	Sensitive (S) or Resistant (R)
Linezolid	S
Kanamycin	S
Amikacin	S
Ofloxacin	S
Moxifloxacin	S
Rifampicin	R
PAS	S
Ethambutol	S
Ethionamide	S
Capreomycin	S
Isoniazid	R
Pyrazinamide	S

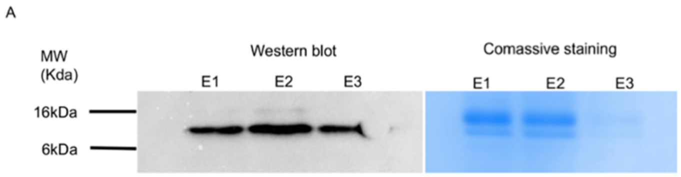

B

Annotated Sequence	Molecular weight	Experimental weight
MHHHHHHENLYFQGGFGcNGPWSEDDIQcHNHcK	4222.7	4222.7
mHHHHHHENLYFQGGFGcNGPWSEDDIQcHNHcK	4238.7	4238.7
SIKGYKGGYcAR	1359.7	1359.7
GGFVcKcY	990.4	990.4

Figure S1. Confirmation of recombinant NZX. (**A**) NZX protein bands observed via western blot and commissive staining obtained from *E. coli* BL21 lysate. One distinct protein band was detected in the first three elution samples detected by western blot using anti-his secondary antibody. A band at the same molecular weight was seen for commissive staining. (**B**) Identity of NZX was confirmed by mass spectrometry from a pooled sample (elution1, 2 and 3).

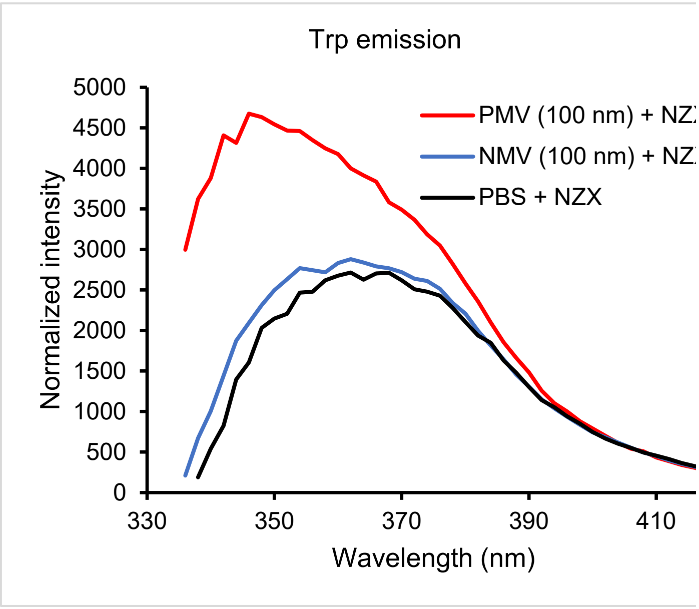

(A)

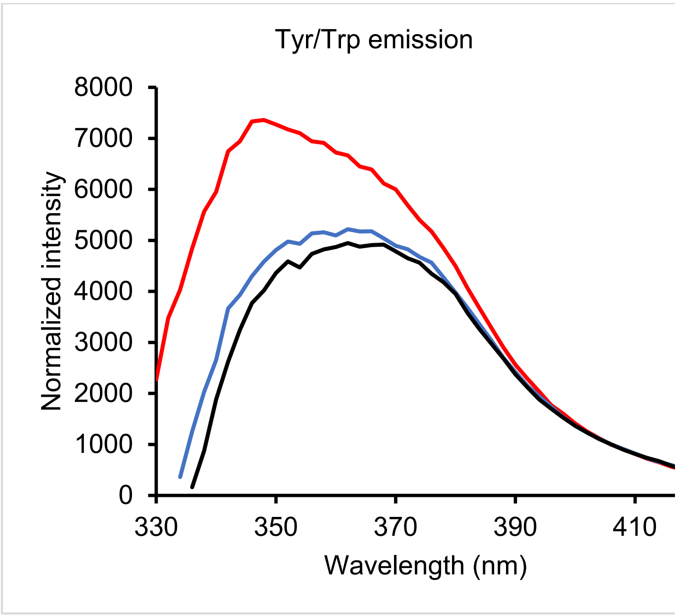

(B)

Figure S2. The non-normalized spectra of intrinsic Trp (**A**) Trp + Tyr (**B**) and emission spectra of the peptide when interacting with the LUV at pH 7.4.

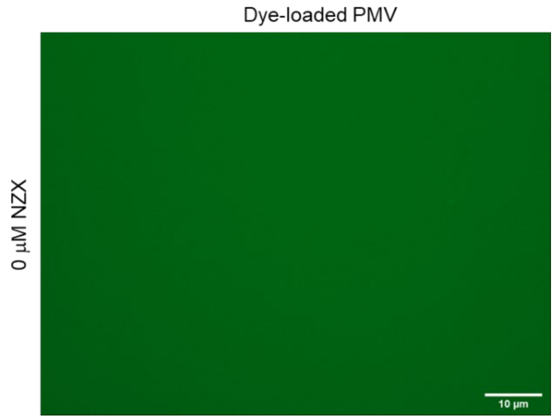

Figure S3. A control image with liposomes without the peptide.
